# Teacher and pupil perspectives on the use of Virtual Field Trips as physically active lessons

**DOI:** 10.1186/s13104-015-1698-3

**Published:** 2015-11-25

**Authors:** E. Norris, N. Shelton, S. Dunsmuir, O. Duke-Williams, E. Stamatakis

**Affiliations:** Department of Epidemiology and Public Health, University College London, 1-19 Torrington Place, London, WC1E 7HB UK; Department of Clinical, Educational and Health Psychology, University College London, 26 Bedford Way, London, WC1H 0AP UK; Department of Information Studies, University College London, Foster Court, London, WC1E 6BT UK; Charles Perkins Centre, University of Sydney, Sydney, Australia; Exercise and Sport Sciences, Faculty of Health Sciences, University of Sydney, Sydney, Australia

**Keywords:** Virtual Field Trips, Physically active lessons, Qualitative, Schools, Children

## Abstract

**Background:**

Virtual Field Trips (VFTs) are emerging physically active lessons that combine curriculum content with globe-based movement using interactive whiteboards. No research has yet examined the acceptability of these sessions by target users. This study aimed to (1) assess current physically active lesson teaching practices, (2) assess teacher attitudes towards VFTs and (3) investigate pupil perceptions of VFTs.

**Methods:**

Data was collected from teaching staff interviews (n = 12) and three elementary school pupil focus groups (k = 3, n = 18), with all participants provided with a sample VFT session. Thematic analysis was used to analyse data.

**Results:**

Teachers described VFTs as a flexible teaching tool, allowing inclusive learning across abilities and a range of taught subjects. They stressed a packed curriculum may make delivering VFT sessions problematic and warned that some teachers may be resistant to their use of technology. Pupils enjoyed the ability to move in the classroom and the ability to share a new teaching experience with their peers.

**Conclusions:**

This work suggests positive attitudes towards VFTs as novel, physically active lessons and identifies potential teacher concerns for consideration in forthcoming intervention planning. Future experimental work will assess if these attitudes persist during longitudinal exposure to VFTs.

## Background

Today’s children spend around 7–8 h a day in sitting (sedentary) behaviours [1]. This is despite current guidelines recommending children to minimise their sedentary time and engage in 60 min or more of moderate or vigorous physical activity (MVPA) [2]. A recent English study found only 24 % of children met these MVPA guidelines, including 19 % girls and 29 % boys [3]. Evidence has shown sedentary behaviour in children to have negative effects on cognitive performance [4] and poorer mental health [5]. With physical activity (PA) [6] and sedentary behaviour (SB) found to track from childhood into adulthood [7], it is clear that both good and bad habits developed in childhood are likely to persist into adulthood. Hence, early intervention to reduce SB and increase PA is imperative [8].

Schools provide an ideal environment to improve children’s PA, as they allow frequent access to children in an inclusive way and over regular periods of time [9]. A wide range of interventions have improved PA across the school day, including active travel [10], playtime [11, 12] and after-school activity [13]. Research has found that school-based PA can improve [14] or not compromise children’s academic performance [15].

Teachers consistently report a lack of time as a barrier for PA interventions [16, 17]. Multiple demands for academic, physical and social outcomes are present in schools [18], often making it challenging for teachers to integrate PA into their busy curriculum [19]. As such, current research is investigating the potential of ‘physically active lessons’: integrating movement within curriculum teaching [20]. Examples of programmes include Physical Activity Across the Curriculum [21] and Take 10! [22]: integrating short-bursts of movement into Maths and English teaching. Increases in PA and educational outcomes have been found with physically active lesson programmes [20, 21]. These findings along with neurological evidence finding positive associations between PA and mental function in children [23], provide strong support for the inclusion of regular, physically active lessons in typical teaching.

Despite evident positive effects of school PA interventions, implementation rates are relatively low [17, 21]. Previous qualitative interviews and process evaluation research has uncovered facilitators and barriers effecting the implementation of school-based PA interventions [17, 24, 25]. The most commonly cited barrier is lack of time: with teachers perceiving PA interventions as difficult to fit in around academic demands [16, 17, 26]. Additional school-level barriers include lack of space for PA, safety concerns, high staff turnover and curriculum clashes with PA interventions [27, 28]. Child and teacher interest towards PA and the intervention itself have also been shown to be important mediators of PA intervention behaviour change [29]: with greater intervention enthusiasm likely in those already active [16, 27, 28, 30]. In order to maximise implementation, it is essential that institutional and individual barriers at all stages of intervention are anticipated and accounted for [31] pilot work and collaborative development between researchers and teachers can help identify potential barriers and minimise attrition for newly developed interventions [32].

Virtual Field Trips (VFTs) may be a viable format of physically active lesson. Originally developed as sedentary, computer-based activities for university teaching [33], VFTs allow individuals or classes to explore virtual maps and landmarks alongside educational content and media [34]. A recent feasibility study tested 10-min VFT sessions in primary schools [35]. These use interactive whiteboards, a pervasive form of classroom technology found in over 70 % of UK classrooms [36]. Via Google Earth-based software, these sessions allow pupils to simulate movements around the world featuring questions and information included according to teaching objectives. Pupils stand throughout these VFT sessions and complete on-the-spot movements simulating actions at or travelling to different locations. This feasibility study showed significantly increased light, moderate and vigorous activity in an active VFT session compared to a sedentary version [35]. Although the study enabled practical VFTs considerations to be identified and addressed in future work, it did not allow documentation of perceived barriers and facilitators to VFT use in teachers and pupils. A qualitative study was hence devised to evaluate the appropriateness, strengths and weaknesses of VFTs in teacher and pupil populations prior to larger-scale intervention work [37, 38].

This study aimed to: (1) assess current physically active lesson teaching practices, (2) assess teacher attitudes towards physically active VFTs after a sample session and (3) investigate pupil attitudes of physically active VFTs after a sample session. The research questions for this study were: (1) To what extent are physically active lessons present in UK teaching practice? (2) What are the perceptions of teachers towards VFTs as physically active lessons? (3) What are the perceptions of pupils towards VFTs as physically active lessons?

## Methods

### Design

Teacher semi-structured interviews and pupil focus groups were carried out. Children are less familiar with one-to-one discussion with an adult [39], hence focus group methodology was used to obtain the views of multiple children in a more relaxed environment. The interviewer (EN) acted as a ‘moderator’ to facilitate comfort, ensure a focused discussion contributed to by all and to seek clarification of unclear points [40]. The Consolidated criteria for Reporting Qualitative studies (COREQ) checklist was followed [41].

### Participants

Convenience sampling in the London area was used for both teacher interviews and pupil focus groups. Teachers were recruited during the 2013/14 school summer holidays via personal contacts and social media. Pupils were recruited during the Autumn 2014 school term via direct enquiries to schools, with no pupils from the same schools as interviewed teachers. No teachers or pupils had experienced VFTs prior to the interview and pupil focus group sessions. Discussions were organised until saturation of ideas was judged as reached by the researcher (EN) [42].

### Instrumentation

An interview script of open-ended questions was developed for teacher interviews and pupil focus groups to ensure standardised enquiry. This featured opening questions exploring perceptions on child school-based physical activity and physically active lessons. A demonstration of a developed Olympic-themed VFT was then provided. Full details on the development of this VFT can be found in the pilot study paper [35]. A 10-min version of the pilot study VFT was used in this session, developed by the lead researcher (EN). Teachers and students visited three locations from the London 2012 Olympics before visiting Rio: the site of the 2016 Olympics. Activities were integrated in travel between locations e.g. simulating swimming across the Atlantic Ocean and in videos at locations e.g. cycling around the London Velodrome. The VFT used in this study was a shortened version of that used in the pilot study [35] and was provided using Google Tour Builder. This is a browser-based modification of Google Earth, which allows users to plot journeys via different locations around the world [43]. Questions then assessed the potential benefits and weaknesses of VFTs as physically active lessons. Interviews were recorded using an Olympus DM-450 Dictaphone.

### Procedure

Teacher interviews were held at a time and place convenient to each participant, typically in their home. Pupil focus groups were held at schools in vacant classrooms. Seating was arranged in a circle and children were allowed to choose their own seat [39]. Focus groups were run by the lead researcher (EN), with no teachers present to prompt honest responses [40]. The researcher also acted as a ‘moderator’ to ensure a focused discussion contributed to by all pupils [40]. To ensure audible recorded comments, an inflatable globe was passed between pupils to denote the person speaking at that time. The researcher made field notes during teacher interviews and pupil focus groups.

**Fig. 1:**
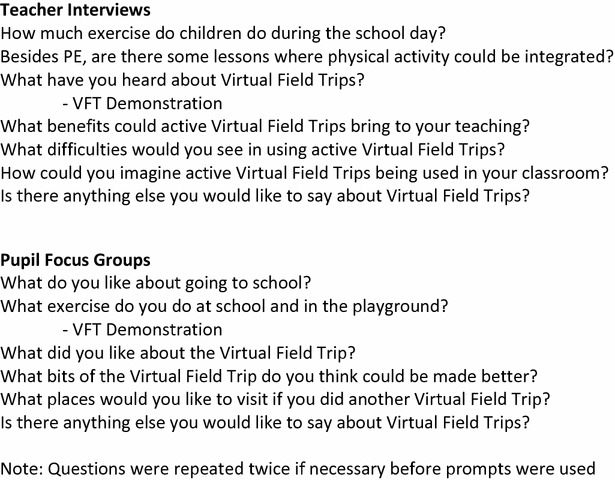
Teacher interview and pupil focus group questions

Informed consent forms were signed prior to interview, with pupil consent forms signed by parents. Teacher interviews lasted between 20 and 60 min and pupil focus groups lasted between 40 and 60 min. Questions first assessed the interviewee’s attitudes towards school-based physical activity. A demonstration of a VFT was then provided by the researcher on a laptop (teacher interviews) or an interactive whiteboard (pupil focus groups). Questions then assessed the potential benefits and weaknesses of VFTs as physically active lessons (Fig. 1). Ethical approval from University College London was granted for both components of this research.

### Data analysis

All interviews were audio recorded and transcribed verbatim on the day of the interview using Express Scribe software. Thematic analysis was used to analyse transcripts, with related quotes clustered to provide raw data themes [44]. An inductive approach was used to allow themes to emerge directly from the data [45]. Statements were read and re-read by two research team members, with emerging themes noted before being clustered into related concepts [44]. The number of participants reporting each theme was recorded.

## Results

Themes are organised according to each research aim and presented in order of diminishing prevalence.

### Teacher interviews

Twelve teachers were interviewed, with ten working in primary-schools (ages 4–11) and two working in secondary schools (ages 11–18). Eleven interviewees were female, one interviewee was a primary school head teacher, one was a PE co-ordinator and two worked primarily with Special Educational Needs (SEN) children. Collectively, the participants held 62 years’ experience working in schools.

### Current physically active lesson teaching practices

Many interviewees (n = 8, 66.7 %) described physical activity in taught lessons as “becoming more the norm” and routinely taught in modern teacher training. Incorporating PA was also described as representative of engaging teaching practice by some participants (n = 4, 33.7 %), “If teachers aren’t doing that then they’re boring teachers”. There was evident variability in use of physically active lessons, with teachers (n = 4, 33.7 %) describing use of PA lessons according to perceived group learning styles. “I think it just depends on the children*”.* PA breaks and lessons were mentioned by interviewees (n = 8, 66.7 %) as a de-stressing and focusing technique for children: “it gives you both a bit of a break as well and it just re-jigs their mind and gets them back on task*”*.

### Barriers of physically active Virtual Field Trips

#### (1) Time

All teachers described children as insufficiently active during school hours due to a “packed curriculum” and academic pressures. Movement was stated as often restricted to break and lunchtimes due to academic pressures. For example, P.E. classes were mentioned by some (n = 4, 33.3 %) as often removed in favour of other academic lessons: “PE as well is one of the lessons where if you’ve got something else planned that you need to do then PE is the one that goes*”.* One participant summarised this sentiment: “I think how much time do you have? There’s so much pressure on what they’re learning and the timetable’s so rammed so you can’t fit any more in.” All but one interviewee (n = 11, 91.7 %) stressed that future VFT intervention packages must provide ready-to-use sessions: “anything like this needs to be easy to implement that can be used straight away”. Many (n = 8, 66.7 %) also mentioned a need for clear guidance as to how each VFT session fit into curriculum topics, to help teachers envisage their usage more easily.

#### (2) Resistance to technology

Some interviewees (n = 7, 58.3 %) highlighted that VFTs could receive different reactions from teachers depending on their ICT competencies. They reflected on the attitudes of other colleagues towards interactive whiteboard use: “There’s some staff who rely so heavily on technology… but there’s teachers who are just very old school who don’t even want to use the interactive whiteboards”. This was stressed as a vital consideration to keep in mind during school and teacher recruitment. However no interviewed teachers felt that they themselves would have difficulties using the technology.

A minority of teachers (n = 3, 25 %) queried the suitability of VFT technology to reduce sedentary behaviour: “You could use electrical tools like this but you don’t have to”. They explained how they saw technology itself as a primary cause of child inactivity and that a non-technological alternative may be more appropriate. For example, “we’re using technology to solve a problem that technology has caused”, suggests perception of a cyclical relationship between technology and activity in these interviewees. Some teachers (n = 2, 16.7 %) described how outside activity is valued over class-based movement in their schools. For example “Schools like mine (would say) ‘Take them outside’”.

#### (3) Potential novelty factor

Some interviewees (n = 3, 25 %) identified a potential limited “novelty” factor for the interactive maps and media content in VFTs. Although all teaching staff had not seen the Google Tour Builder used before, some were wary it could become stale after a few lessons. For example: “think the novelty will be there with un-technological children but for those that are used to technology, they’ll be like ‘OK I get it’” Additionally, two teachers (n = 2, 16.7 %) reflected that schools find it difficult to keep up with frequent evolutions in technology: “with technology, nothing impresses them. If anything we’re more impressed because we are behind, children are ahead of us”.

### Facilitators of physically active Virtual Field Trips

#### (1) Flexibility of VFTs as teaching tool

All teachers provided a variety of creative ways that VFTs could be used in their teaching. These ranged from “starters or plenaries’ and as brain breaks to increase children’s attention in the morning or after lunch. Teachers also enthusiastically provided a multitude of topics that VFTs could cover. Common areas included geography and history based themes, such as “With Year 5… the Victorians, Africa, the Aztecs, water, Geography, Earth Sun and Moon in Science, extreme Earth like tsunamis and lightning…”. Some teachers (n = 5, 41.7 %) also mentioned the potential for ‘Maths’ or English-based sessions: “You could also do a story map of a book.” One teacher (8.3 %) also emphasised the potential for VFTs as physical education teaching tools: “this lends itself beautifully to PE teaching… give me something on there that shows me the correct technique… we learn together as we practice it in class.”

Additionally, some (n = 5, 41.7 %) described physically active VFTs as being “cross-curricular” in nature by linking physical activity to other topics: an important feature in the new English National Curriculum [46]. A minority (n = 3, 25 %) also mentioned the opportunity to add a competitive element to VFTs: encouraging group-based challenges to be more active. For example, “You could have competition and a leader board that was topic-based like with the fire (Great Fire of London): ‘who could run away from the fire?’ or ‘Who can escape the plague quickest today?’

### (2) VFTs for inclusive learning

A common theme throughout all teachers was the potential of VFTs as teaching tools to include all pupils in an equal learning environment. The presence of both visual and kinaesthetic elements in active VFTs was appealing to many (n = 6, 50 %) who saw this as “encouraging all types of learning styles to participate in lesson which is really good*”.* Some teachers (n = 5, 41.7 %) also described physically active VFTs as useful to manage behaviour in pupils with attention disorders. Examples of conditions included ADHD, autism, or “those that a general static lesson doesn’t necessarily grasp their attention for long enough”. Teachers reflected on their use of physical activity in lessons especially to cater for these populations: “We do that all the time if any kid with SEN or ADHD, we always have physical activity involved in their lessons and that’s mainly for them!”

### Pupil focus groups

Three focus groups were held, two with Year 4 pupils (n = 12; 8–9 years old) and one with Year 5 pupils (n = 6; 9–10 years old). Nine boys and nine girls participated.

### Experiences of school-based physical activity

All pupils reflected on their enjoyment of school playtime, swimming and extra-curricular physical activity opportunities. They also provided memorable experiences of lessons combining physical activity: “I enjoy when we have Maths and sometimes we go outside and we do activities… have to do charts of the activities.”

### Views on physically active Virtual Field Trips

#### (1) VFTs to share experiences with peers

All students commented on VFTs as an opportunity to have “fun” with their peers. As seen in teacher interview, pupils also suggested the introduction of teams to encourage physical activity competitiveness during the sessions. It was also mentioned that alternation of these teams would allow interaction socialisation with different pupils. For example, “You could have like a weekly group and you could keep changing it round so you get to socialise with other people… and just like try and also get to know them while learning.” Three students (16.7 %) mentioned how VFTs could be used to explore and share countries of their heritage, “I would like to go back to my home country… I’ve heard these really cool stories about this really big volcano there and I would like to see it”.

#### (2) VFT novelty

Pupils indicated familiarity with Google Earth software but described liking the novelty of using their bodies during the lesson and to answer questions. “I liked it… You could move around and use body movements to get picked (to answer a question).” Pupils also discussed how being active made them feel more immersed in the locations of the VFT: “you was like moving your arms, legs and your stomach to actually feel like you’re actually going to that country”.

#### (3) Exertion of VFT physical activity

Some children commented on feeling tired after the demonstration VFT: “You really get tired as you start to travel somewhere…” This may be expected given that this teaching style is innately different to the sedentary style they are used to.

## Discussion

A range of evidence from qualitative interviews and process evaluation work exists evaluating the efficacy of school-based PA interventions. This study extended previous pilot work which trialled VFT technology and outcome measurements, by assessing teachers and pupils perceptions towards physically active VFTs. Despite there being little empirical evidence in the UK [20], teachers reported common use of physically active lessons in their own practice. A range of factors facilitating VFT use were identified. Teachers praised VFTs as inclusive learning tools due to their innate combination of kinaesthetic, audio and visual elements. They also provided a broad range of suggestions for potential VFT sessions, showing big scope for integration across the curriculum. Both teachers and pupils identified a potential for active VFTs to enable challenges between classmates: encouraging pupils to compete and be more active in sessions. This desire for competitiveness in VFT sessions contradicts previous research, finding increased overall PA enjoyment when rivalry is not involved [47].

Various important barriers were identified. Firstly, as with previous school-based PA intervention research; teachers saw a lack of teaching time as a potential barrier for VFT use [16, 17]. To maximise recruitment and fidelity in future research, teacher training must stress research showing increased PA in schools to not compromise academic achievement [15]. VFT interventions must be presented and acceptable to multiple stakeholders such as the head-teacher, teachers, parents and governors [18] to maximise uptake and minimise disruption.

Secondly, although VFTs were described by the researcher as ready-made sessions, some warned of potential resistance in teachers less confident in using technology. This may produce a biased sample as such teachers may hence be less likely to participate in VFT interventions or less likely to complete them as intended. Full training will be provided in future intervention work but this may still be insufficient to encourage some teachers of the merits of active VFTs. Some teachers also queried the use of technology to improve sedentary behaviour, as they described this as the result of children’s increasingly technological lives. It seems a cyclical relationship between technology and activity is observed by these interviewees, which may again prevent VFTs being implemented. These considerations of VFT use replicate the Technology Acceptance Model [48, 49]. Under this model, teachers that perceive VFT technology as useful, easy to use and worthwhile will be more likely to use them. It will hence be necessary in future work to maximise these perceptions in teachers by stressing the practical benefits of VFT technology to teachers, such as its quick set-up time and multi-modal functionality. Finally, teachers predicted a potentially short novelty factor for VFTs. By integrating a variety of media, locations and movements into future VFT programmes, it is hoped that pupil’s perceived enjoyment and novelty will persist. Longitudinal VFT study will assess if these perceptions of novelty remain during regular sessions and whether children become more attuned to being active during VFT sessions.

Methodologies used have some limitations. Firstly, there is mixed evidence for holding child focus groups in school settings and using existing class relationships. Pupils may have been positively prompted to answer to the best of their ability as with typical teaching, or may have conversely felt distracted by existing peer relationships or repressed by school expectations of adult-child hierarchies [50]. Secondly, there are specific issues within teacher methodology used. Interviews were held in a variety of locations, as chosen by each participant to maximise recruitment. As these were held during the school holidays, some were held in the interviewee’s home. This may have led to differences in perceived trust or comfort in the interviewee, compared to more neutral environments [51]. Also, the use of convenience sampling to gain teacher participants via personal contacts and social media approaches may have biased responses. Two teachers taught secondary school age pupils to provide insight into teaching considerations for this age-group, although no secondary aged pupils were interviewed. Finally, teachers took part in a demonstration VFT session with the interviewer. However, use of a VFT by teachers themselves in an actual lesson may have prompted different responses. Future longitudinal intervention work will include a full process evaluation using teacher and pupil data, to enable deep assessment of VFT facilitators and barriers.

## Conclusions

This study provides valuable insight into the perceptions of teachers and pupils of physically active lessons generally, as well as novel Virtual Field Trips as active lessons. Potential VFT barriers identified here will need to be addressed in future longitudinal work during recruitment, training and intervention development. It is clear from this study that although teachers and pupils are receptive to physically activity lessons; the use of classroom technology for interventions must be made transparent and efficacious for maximal uptake.

## Abbreviations

VFTs: Virtual Field Trips
PA: physical activity
